# Supplementary material for: Are carbon emissions trading and green financial instruments synergistic? -Comprehensive quantitative research based on content analysis
Source: PLoS One. 2024 Mar 7;19(3):e0298601. doi: 10.1371/journal.pone.0298601 (PMC10919601; doi:10.1371/journal.pone.0298601)
Supplement: S1 Data — (ZIP) [file pone.0298601.s001.zip › Data/地级市Z_F工作报告.zip!/╣π╢1⁄2/╜╥╤⌠╩╨/└┤╫╘╣1⁄2╓┌║┼í╛┬φ┐╦╩2╛▌═°í┐_2010.docx]

各位代表：

　　现在，我代表市人民政府向大会作政府工作报告，请予审议，并请政协各位委员和其他列席人员提出意见。

　　2009年工作回顾

　　过去一年，全市人民在市委的正确领导下，深入贯彻落实科学发展观，全面实施“一三二”发展思路，攻坚克难，化危为机，全力加快经济社会发展，取得了显著成效。

　　——经济增长目标圆满完成。主要经济指标增速名列全省前茅。全年实现生产总值815.77亿元，比上年（下同）增长16%；万元生产总值能耗下降3%，二氧化硫、化学需氧量分别减排6.25%和2.52%；规模以上工业增加值274.41亿元，增长26.8%；全社会固定资产投资374.72亿元，增长41%；外贸出口25.25亿美元，增长20.4%；社会消费品零售总额340.45亿元，增长28.3%；地方财政一般预算收入28.87亿元，增长29.03%；城镇居民人均可支配收入13169元，增长12%；农村居民人均纯收入5433元，增长10.3%，完成或超额完成了市四届人大四次会议确定的各项目标任务。

　　——项目建设取得重大进展。新增1000万元以上重点项目255个。完成市级重点项目投资172.5亿元，其中省重点项目投资79.6亿元。中石油广东石化炼油项目、中海油粤东LNG一体化项目、中电投广东揭阳储煤配送中心项目成功落户并获准开展前期工作；华润仙安风电场建成投产，关山风电场开工建设，累计建成风力发电装机容量21.32万千瓦；惠来电厂3、4号机组抓紧推进；中广核揭阳核电项目进入可研阶段；完成电网投资10.64亿元。揭阳潮汕机场正式开工，航站楼进入上部土建结构工程施工阶段；率先完成厦深铁路揭阳段征地拆迁任务，大南山隧道提前贯通；建设省养公路139.6公里，其中国道206线市区至丰顺、省道236线市区至池尾等路段建成通车，国道206线曲溪至小坑段、省道335线曲溪至深坑段完成主体工程建设；新（改）建农村公路648公里，全面实现通村公路水泥化目标。

　　——产业竞争实力不断增强。落实扶持企业贷款贴息财政性资金1000万元，促成第二批银企融资协议111.78亿元，全年新增贷款100.15亿元，存贷比提高8.7个百分点。珠海（揭阳）产业转移工业园成功竞得省5亿元扶持资金，首期6.22平方公里控制性详规取得成果并组织实施。新增规模以上工业企业452家，限额以上商业企业350家，500万元以上工业项目276个。《揭阳市沿海重化产业带发展规划》、《揭阳市旅游发展总体规划》编制工作扎实推进，《揭阳市大型专业市场和现代物流发展规划》顺利实施。第三产业比重比上年提高1个百分点。50家企业与51家高校、科研机构签订59项产学研合作协议；新增中国高新技术企业11家、省民营科技企业11家，省级企业技术中心4个、省级工程技术研发中心5个、省级专业镇2个、省级标准化技术委员会（TC）1家；新增中国驰名商标1件，省著名商标10件，省名牌产品8个，累计注册商标25770件。

　　——统筹城乡发展力度加大。去年是揭阳规划年，《揭阳市城镇体系规划》、《揭阳市城市总体规划》、《揭阳市土地利用总体规划》、《揭阳市渔湖新区控制性详细规划》、《揭阳市空港经济区总体规划》、《揭阳港总体规划》、《揭阳市海洋功能区划》修编（编制）工作取得重大成果。市区建设两个“30亿工程”扎实推进，新阳东路、晓翠北路、莲花大道北路、市区污水处理厂、东湖公园、文化公园等市政基础设施建成投入使用，省级卫生城市创建工作和 “三旧”（旧城镇、旧厂房、旧村庄）改造工作全面铺开，城市功能逐步配套，市容市貌明显改观。水环境综合整治工作深入开展，县（市、区）8个污水处理厂建设进展加快。农村信息化综合信息服务试点工作初见成效。214个试点村改制为社区居委会。全面实施扶贫开发“规划到户、责任到人”，抓好归难侨安居工程收尾工作，农村残疾人脱贫脱困工作取得明显成效。除险加固水库21宗，累计解决农村饮水不安全人口67.13万人。建设标准农田6.02万亩，利用园地山坡地开发补充耕地4.96万亩。完成造林1.33万公顷，大北山森林公园晋升为国家级森林公园并成功列为省大型重点旅游扶贫项目。保障发展用地工作水平不断提高，土地矿产违法现象得到有效整治。

——社会建设取得明显进步。筹措基础教育资金14亿元，新（扩）建校舍113.68万平方米，10所新建高（职）中有9所完成一期建设任务。参加高考人数和上线人数分别达到3.75万人和2.72万人。中小学教师工资福利待遇“两相当”基本落实，1533名代课教师经招考转为公办教师。成功举办首届特色文化节和第五届运动会，新增省级非物质文化遗产16项，新建基层文化设施220个，揭阳楼、市图书馆、市广电中心、体育馆、学宫修缮等重点工程建设进展顺利。甲型H1N1流感疫情得到有效防控，新型农村合作医疗惠及更多农民群众。城镇新增就业3.27万人，登记失业率3.13%；农村劳动力参加职业技能培训5.11万人，转移就业11.93万人。全市享受城乡低保12.6万人，参加基本医疗保险53.04万人、企业养老保险19.77万人，实现应保尽保。基本建立廉租住房保障制度。对口援建汶川卧龙镇工作进展顺利。全市人口出生率11.32‰，自然增长率6.33‰，政策生育率93.04%，完成省下达人口控制指标。规范市场经济秩序，清理无照经营11725户。财税征管得到加强，率先在全国成功开发建安业和房地产业税源控管系统。食品药品监管力度加大，安全生产四项主要指标全面下降，社会治安状况持续好转。去年再次被评为全省综治工作优秀市，榕城区被评为全国平安建设先进区。外事侨务、民族宗教等其他事业不断进步。

　　——政府自身建设得到加强。主动接受人大、政协监督，办理省、市人大代表建议53件，政协委员提案建议106件。建立政府常务会议学法制度，全面实行行政首长问责制，依法行政水平不断提高。抓好新一轮政府机构改革，加快转变政府职能。取消和停止市直行政事业性收费34项，年减轻社会负担8000多万元。行政效能不断提高，市直电子监察系统实时监察行政审批业务16477件，提前办结率99%.加强审计监督和行政监察，认真落实厉行节约八项要求，实现“五个零增长”和“四个减半”目标。

　　各位代表，过去一年取得的成绩来之不易。这是省委省政府和市委正确领导、市人大政协大力支持、全市人民共同奋斗的结果。在此，我谨代表市人民政府，向全市各条战线的干部群众，向所有关心支持揭阳发展的同志们、朋友们和海内外乡亲们，表示衷心的感谢！

　　在看到成绩的同时，我们也清醒地认识到，我市经济社会发展仍存在不少困难和问题，主要是：产业发展水平低，加快发展压力大；城市化水平不高，城市功能仍不完善；社会事业相对滞后，人民生活质量有待提高；发展环境仍需改善，社会管理亟待加强。我们将加倍努力，采取有效措施，认真解决这些存在问题。

　　2010年工作安排

　　2010年是全面实现“十一五”规划目标的决胜之年，也是推动揭阳科学发展、跨越发展最为关键的一年。做好今年政府工作，事关大局，影响深远，必须科学谋划，精心安排，狠抓落实。今年政府工作的总体要求是：以邓小平理论和“三个代表”重要思想为指导，深入贯彻落实科学发展观，认真贯彻党的十七大、十七届四中全会、省委十届六次全会、粤东地区现场会、粤东地区工作会议和市委四届七次全会精神，全面实施“一三二”发展思路，围绕转变经济发展方式这个核心，突出“三加快一确保”这个重点，狠抓现代产业体系建设，狠抓自主创新，狠抓扩大内需，狠抓社会建设，狠抓民生保障，以大项目带动大发展，以工业化促进城市化，强势推进跨越发展，努力实现赶超进位、后来居上，力拔粤东“五年大变化”头筹。

　　主要预期目标是：生产总值增长20%左右，力争突破1000亿元大关；单位生产总值能耗下降1.8%，二氧化硫、化学需氧量分别削减16.67%和6.98%；规模以上工业增加值增长38%；全社会固定资产投资增长40%以上；外贸出口增长15%；社会消费品零售总额增长28%；居民消费价格涨幅控制在3%左右；地方财政一般预算收入增长30%左右；城镇登记失业率控制在3.5%以内；人口自然增长率控制在6.8‰以内；城镇居民人均可支配收入增长13.5%，农村居民人均纯收入增长8%.

实现上述目标，必须突出抓好以下五方面工作：

　　一、狠抓大项目，拉动大投资，力争率先实现跨越发展

　　千方百计引项目，聚精会神抓投资，全力以赴抓好12个重大项目建设，加快推进192个市级重点项目，全面梳理和落实粤东会议确定的382个重点项目，做到“上马一批、建成一批、引进一批、储备一批”，为率先实现跨越发展注入强大动力。

　　突出抓好能源重点项目建设。举全市之力推进中石油广东石化炼油项目、中海油粤东LNG一体化项目、中电投广东揭阳储煤配送中心项目前期工作，争取国家尽快核准并开工建设。抓紧建设惠来电厂3、4号机组及送出工程。完成中广核揭阳核电项目可行性研究，争取列入国家新能源发展规划。继续抓好华润热电联产等重点能源项目的跟踪落实。加快城乡电网改造，争取揭西龙颈水库抽水蓄能项目列入南方电网建设计划。

　　全力推进交通基础设施建设。完成揭阳潮汕机场航站楼土建结构和厦深铁路揭阳段土建工程并做好设备安装工作，加快推进汕揭、潮揭高速公路建设项目，争取动工建设潮州至惠州和汕头至河源高速公路揭阳段、揭东至惠来高速公路，抓紧完成国道206线曲溪至小坑段、省道335线曲溪至深坑段配套工程，改造市区北平交至揭阳高新区、国道324线池尾至葵潭段等道路，推进亿吨产业大港建设，加快形成海陆空立体交通格局，打造区域性交通枢纽城市。

　　加强水利基础设施建设。全面完成城乡水利防灾减灾和病险水库除险加固工程建设任务，抓紧推进三洲拦河闸后续项目等重点水利工程。争取尽快启动韩江引水工程。围绕重大项目落户建设，积极实施惠来西水东调计划，加快建设赤岑水闸、邦山水闸等水利设施，为发展大工业提供水资源保障。

　　高度重视项目策划引进工作。加强产业和政策研究，尤其是对石化产业链的研究，积极做好项目策划、论证、储备、引进工作。加大招商引资力度，争取更多大型国有企业、跨国集团、民营骨干企业到揭阳落户发展。继续实施“乡贤回归”工程。抓好珠海（揭阳）产业转移工业园、大南海国际石化综合工业园、空港经济区等园区建设，积极承接国内外产业转移。

　　二、实施大创新，谋求大转型，推动经济又好又快发展

　　坚持抓创新调结构促转型，深入扩内需拓市场强外贸，推动经济持续快速发展。

　　大力推进自主创新。深入实施知识产权战略纲要，加快建设创新型揭阳。综合运用财税、土地、环保等调控手段，建立健全有利于自主创新的体制机制。加强各类人才培养引进，建设创新型人才队伍。鼓励企业与高等院校、科研机构共建重点技术研发机构，联合开展技术攻关，提高产学研合作水平。加强行业公共技术平台和创新服务体系建设，支持企业开发核心技术，参与制定国家、地方和行业标准，创建品牌商标，增强企业核心竞争力。力争今年有3—5家企业列入省“自主创新100强”，新增6家中国高新技术企业、1个国家级标准制定工作组、12家省级民营科技企业、3个省级企业技术中心、1个省级工程技术研发中心。

　　优化提升传统产业。加强省市共建支柱产业建设，确保国家不锈钢制品质量监督检验中心按期投入使用，争取服装产品省级检验站升格为国家检验中心，不断扩大区域品牌影响力，做大做强五金不锈钢、纺织服装、玩具鞋类等传统优势产业。深化国有企业改革，落实扶持企业发展政策措施，加大技术改造支持力度，激活和扩大民间投资；继续推进“三个新增100”，抓好拟上市公司指导工作，力争今年揭东县、惠来县、榕城区各有1家以上、普宁市有2家以上企业上市，加快打造12条经济大船，提升产业发展层次和水平。

　　加快发展现代产业。充分发挥重大项目带动作用，加快形成以重化产业和现代服务业为“双核心”的现代产业体系。大力发展电力能源、石油化工、装备制造、电子信息等战略产业和先进制造业，争取一批项目列入广东现代产业“500强”。抓好10大国际商贸城建设，着力提升餐饮住宿、旅游休闲、家政养老等传统服务业，突出发展金融保险、现代物流、信息咨询、服务外包、总部经济、文化创意等高端服务业。争取成立揭阳市榕城农村商业银行和揭西农村商业银行，积极稳妥地做好城市信用社退市工作，深化银企合作关系，提升金融服务水平，发展壮大金融产业。力争服务业增加值占生产总值比重达到35.7%以上。

搞活搞旺内外经贸。提升国际玉器节、纺织服装交易会、五金不锈钢制品博览会的档次和水平，增强影响力和辐射力。落实家电下乡、以旧换新等扩大消费政策措施，繁荣活跃商贸流通。办好揭阳（武汉）产品展销会，推动揭货“北上西进”。加强区域经济协作，主动融入珠三角，对接海西区。巩固和发展东盟、中东等新兴市场，积极开拓北美、欧洲等国际市场，办好赴美展销活动，鼓励企业创建出口品牌，申报进出口经营权，推动企业转型升级，促进外贸出口较快增长。扩大紧缺物资进口，积极引进先进适用技术。提高吸收外资水平，确保实际利用外资有所增长。抓好揭阳港一类口岸和揭阳潮汕机场航空口岸申报建设。筹办好广东省第三届粤东侨博会暨揭阳市第二届特色文化节。

　　三、服务大工业，加快一体化，建设宜业宜居宜游城乡

　　适应办大工业的新要求，以提高城镇化水平为重点，以建设宜居城乡为抓手，力促城乡一体化发展有新突破。

　　建设宜居城乡。充分发挥规划的引领和调控作用，争取《揭阳市城镇体系规划》尽快获省审批并组织实施，抓紧完成《揭阳市城市总体规划》修编工作，促进城镇协调有序发展。强化项目建设报批管理，深入开展土地卫片执法检查，严厉打击违法违规建设行为。加快推进市区建设两个“30亿工程”，开发建设50平方公里新城区，继续落实各县（市、区）每年筹资1亿元以上投入城区市政建设，增强城镇辐射带动功能，促进产业和人口集聚发展，力争今年城镇化率达到60%.加强城市精细化和网格化管理，深入开展创建省级文明城市和卫生城市活动，净化、美化、亮化城市环境，提升城市形象和品位。充分发挥住房公积金支持住房消费的作用，促进房地产市场健康发展。加强规划管理，用好用足政策，全力推进“三旧”改造和建设，力争“一年一大步、两年见成效、五年大变样”。

　　建设生态城乡。强化人口计生管理，稳定低生育水平，确保普宁市、惠来县按期转化升类。扎实推进集体林权制度改革，抓好绿道网规划建设，保护开发黄岐山森林公园和紫峰山风景区，按期实现“绿化揭阳大地”目标。全面完成《揭阳市土地利用总体规划》和《揭阳市海洋功能区划》修编。落实耕地占补平衡和总量动态平衡，增强保障发展用地能力。落实节能减排措施，深化水环境综合整治，加快县（市、区）18个污水处理厂和8个垃圾填埋场建设，切实提高环境容量和承载能力。

　　建设平安城乡。加强应急处置管理，严格食品药品和产品质量监管，狠抓安全生产综合整治，保障人民群众生命财产安全。落实领导包案、公开接访等信访制度，加强镇街综治信访维稳中心建设，及时排查化解社会矛盾，切实提高司法公正和法律服务水平。实行以居住证为核心的“一证通”制度，深入开展十大专项整治行动，严厉打击“两抢一盗”、贩毒吸毒、制假售假等违法犯罪活动，强化社会治安源头综合治理，创造安居乐业的社会环境。

　　建设新农村。加大农业基础设施投入，推进现代标准农田建设，力争全年改造低产田6万亩以上。认真落实强农惠农政策措施，积极实施科技兴农和旅游兴农工程，大力发展效益农业、生态农业和特色农业，加强农民专业合作组织建设，完善农村市场体系，提升农业产业化经营水平，促进农业增效、农民增收。深入实施“规划到户、责任到人”扶贫开发，力争今年解决农村饮水不安全人口38.8万人，实现脱贫2.5万户。加强农村公共设施建设，抓好生活污水净化处理工程示范推广，大力发展沼气，改善农村生态环境，建设社会主义新农村。

　　四、围绕大和谐，着力惠民生，全面加快社会事业发展

　　高度重视民生民计，统筹推进各项社会事业，让人民群众共享改革发展成果，促进社会和谐、文明进步。

　　优先发展教育事业。继续落实教育资金筹措、校舍建设和提高师生素质三项任务。调整中小学校布局，整合提升城乡教育资源，促进义务教育均衡发展；加快普及高中阶段教育，扩大中职、技工教育办学规模，优化高（职）中教育阶段结构；加强高校规划建设，筹办石化学院；重视学前教育、特殊教育和民办教育，形成各类教育事业协调发展的良好格局。完成市卫校新校区建设。力争全年新（扩）建学校121所，义务教育规范化学校达到60%，高中阶段毛入学率达到70%.做好义务教育学校绩效工资实施工作，全面落实中小学教师工资福利待遇“两相当”，抓紧解决代课教师“代转公”遗留问题。

　推进文化大发展。深化文化体制改革，完成市级有线广电网络、新华书店、电影公司转企改制和重组工作。加快在建文化工程建设，确保揭阳楼、体育馆、市图书馆建成交付使用。启动市文化广场项目建设和学宫二期、丁日昌故居修缮工程。完善县、镇、村文化设施，健全公共文化服务体系。加强历史文化名城保护开发，积极申报国家和省级非物质文化遗产，振兴潮剧事业，传承发展地方特色文化。繁荣文学艺术、新闻出版、影视传媒和社会科学，丰富人民群众精神文化生活。

　　提高医疗卫生保障水平。深化医药卫生体制改革，建立健全覆盖城乡居民的基本医疗卫生制度。推进公立医院改革试点工作，抓好基本医疗保障、药品供应保障、城乡医疗服务、公共卫生服务体系建设，确保新型农村合作医疗参合率达到99.2%，60%政府举办的基层医疗卫生机构实施基本药物制度，实行零差率销售基本药物。加强重大流行疫病防控工作。推进中医药“三名”（名院、名科、名医）和“三进”（进农村、进社区、进家庭）工程，建设中医药大市。

　　加强就业服务和社会保障。完善就业服务体系和就业援助制度，切实解决企业招工和社会就业难题，力争城镇新增就业3.2万人，农村劳动力参加职业技能培训5.5万人，转移就业12万人。坚持依法治税理财，按序时均衡组织收入，集中财力保障民生支出需要。做好企业养老保险、城镇职工基本医疗保险市级统筹工作，全面推进医疗保险城乡一体化管理。抓好揭东县新农保试点工作。认真落实城乡最低生活保障制度，充分发挥红十字会、慈善总会等福利机构的作用，积极做好赈灾救济工作，加快市社会福利中心建设步伐，完成廉租住房建设任务，保障困难群众基本生活。

　　大力发展其他社会事业。巩固“双拥”模范城创建成果，做好国防动员、征兵、人民防空工作。结合广州亚运会开展各项体育活动，增强人民体质。抓好市新体校规划建设。加强物价监管。提高统计服务质量。发展老龄、妇幼和残疾人事业，做好外事侨务、民族宗教、气象地震、档案史志等工作。全面完成对口援建汶川卧龙镇任务。

　　五、构筑大保障，推动大发展，努力开创政府工作新局面

　　围绕经济社会发展大局，着力加强政府自身建设，为完成全年目标任务提供坚强的保障。

　　打造学习型行政机关。完善市政府常务会议学法等学习制度，带动各级行政机关形成浓厚的学习氛围。加强公务员教育管理监督，深入开展感恩奉献和忠诚教育活动，努力建设一支人民满意的公务员队伍。

　　打造服务型行政机关。全面完成市县政府机构改革和事业单位分类改革，认真抓好乡镇政府机构改革、强县富镇事权改革和第四轮行政审批制度改革，推动政府职能由“管理型”向“服务型”转变。整合电子政务资源，推进网上审批和并联审批，切实提高办事效率和服务水平。

　　打造高效型行政机关。强化行政执行力，完善行政问责制，对重点项目、主要经济指标、产业转型升级、城市化和城镇化建设、社会民生建设等五项工作实行插“红旗”制度，坚决兑现奖惩措施。

　　打造法制型行政机关。深入开展法制城市创建活动，严格依法行政，自觉接受人大的法律监督和政协的民主监督，认真办好人大代表议案建议和政协委员提案建议，广泛听取各民主党派、人民团体的意见和建议，充分发挥政府顾问、专家咨询组的作用，广听民声，广纳民意，广集民智，不断提高科学化、民主化和法制化决策水平。

　　打造廉洁型行政机关。加强机关事务管理，严格控制一般性支出。认真落实党风廉政建设责任制，强化审计监督和行政监察，注重从源头上预防和治理腐败，以良好的政风带行风促民风。

　　科学编制“十二五”规划。围绕“五年大变化、十年大发展”的目标要求，精心组织编制“十二五”规划，为新一轮大发展提供科学指导。

　　各位代表！新的一年，我们肩负着更加神圣而艰巨的任务。让我们在市委的坚强领导下，同心同德，开拓进取，扎实工作，为建设富民强市、和谐文明新揭阳而努力奋斗！
